# NIEHS Celebrates 50 Years of Environmental Health Research at the NIH

**DOI:** 10.1289/ehp.1511015

**Published:** 2016-01-01

**Authors:** Linda S. Birnbaum

**Affiliations:** National Institute of Environmental Health Sciences and National Toxicology Program, National Institutes of Health, U.S. Department of Health and Human Services, Research Triangle Park, North Carolina, USA

On 1 November 2016 the National Institute of Environmental Health Sciences (NIEHS) will celebrate its 50th anniversary, five decades after the U.S. Surgeon General announced the establishment of the Division of Environmental Health Sciences at the National Institutes of Health (NIH). Today, from its home in Research Triangle Park, North Carolina, the NIEHS funds more than $750 million in research each year to discover how the environment influences human health and disease.

It is my honor and privilege to serve as the NIEHS director during this significant milestone in the institute’s history, and I see it as an opportunity to highlight the improvements to public health that have resulted from environmental science research. I also want to bring together health researchers supported by the NIEHS, and the NIH as a whole, for networking and collaboration. I’m really excited about all the scientific and public outreach activities we’ve planned for the 2016 anniversary year.

This month the NIEHS will hold an oral history event featuring alumni and retirees who will share their reflections on scientific progress, professional experiences, and personal memories at the institute. We’ll also initiate a time capsule and begin collecting nominations for items to fill it so that we can share our 2016 NIEHS research and culture with future staff and science historians.

We’ll host several distinguished lectures at the NIEHS and hear from top scientists, including Gina Turrigiano, Gerard Karsenty, Myles Brown, and Jeff Gordon. All these lectures will be open to the public and webcast live from our website at http://www.niehs.nih.gov.

The NIEHS will partner with the Society of Toxicology in July for a day-long symposium on technological advances, and with the Endocrine Society in September for a three-day workshop on endocrine disruptor research.

A Women’s Health Awareness event at North Carolina Central University, a public forum at the Research Triangle Foundation, and a Science in the Cinema program at Marbles Kids Museum in downtown Raleigh are just a few ways the NIEHS will engage the communities surrounding Research Triangle Park to share information about environmental health and the value of our research.

On the anniversary day of 1 November 2016, hundreds of research partners, grantees, and public health officials will join institute staff and alumni for a very special program celebrating the history, scientific advances, and public health contributions resulting from the unique and prevention-focused research supported by the NIEHS.

Finally, in December, for the first time ever we’ll bring together at once all our grant-funded research center directors and their key scientific staff from across the United States for a lively exchange of research findings, methods, and community engagement practices.

A full calendar of events is posted on the NIEHS website, and I hope our friends and partners will plan to join us often. We’ll also be posting fun and interesting photos and recordings from the past 50 years.

We hope you’ll take the opportunity in 2016 to tell someone you know about the NIEHS and what it has meant to you. I’d love to hear your stories, and I’ll add them to my own, which began 36 years ago when I started my federal research career as a senior staff fellow at the NIEHS.

